# Postoperative spinal infection mimicking systemic vasculitis with titanium-spinal implants

**DOI:** 10.1186/1748-7161-6-20

**Published:** 2011-09-13

**Authors:** Vasileios I Sakellariou, Erato Atsali, Konstantinos Starantzis, Chrysanthi Batistaki, Triantafyllia Brozou, Panayiotis Pantos, Konstantinos Stathopoulos, Konstantinos Soultanis

**Affiliations:** 1 First Department of Orthopaedics, School of Medicine, University of Athens, Attikon, University General Hospital, Chaidari, Greece; 2 Third Department of Paediatrics, School of Medicine, University of Athens, Attikon, University General Hospital, Chaidari, Greece; 3 Second Department of Anaesthesiology, School of Medicine, University of Athens, Attikon, University General Hospital, Chaidari, Greece

## Abstract

**Background:**

Secondary systemic vasculitis after posterior spinal fusion surgery is rare. It is usually related to over-reaction of immune-system, to genetic factors, toxicity, infection or metal allergies.

**Case Description:**

A 14 year-old girl with a history of extended posterior spinal fusion due to idiopathic scoliosis presented to our department with diffuse erythema and nephritis (macroscopic hemuresis and proteinuria) 5 months post surgery. The surgical trauma had no signs of inflammation or infection. The blood markers ESR and CRP were increased. Skin tests were positive for nickel allergy, which is a content of titanium alloy. The patient received corticosteroids systematically (hydrocortisone 10 mg) for 6 months, leading to total recess of skin and systemic reaction. However, a palpable mass close to the surgical wound raised the suspicion of a late infection. The patient had a second surgery consisting of surgical debridement and one stage revision of posterior spinal instrumentation. Intraoperative cultures were positive to Staphylococcus aureus. Intravenous antibiotics were administered. The patient is now free of symptoms 24 months post revision surgery without any signs of recurrence of either vasculitis or infection.

**Literature Review:**

Systemic vasculitis after spinal surgery is exceptionally rare. Causative factors are broad and sometimes controversial. In general, it is associated with allergy to metal ions. This is usually addressed with metal on metal total hip bearings. In spinal surgery, titanium implants are considered to be inert and only few reports have presented cases with systemic vasculitides. Therefore, other etiologies of immune over-reaction should always be considered, such as drug toxicity, infection, or genetic predisposition.

**Purposes and Clinical Relevance:**

Our purpose was to highlight the difficulties during the diagnostic work-up for systemic vasculitis and management in cases of posterior spinal surgery.

## Background

Systemic vasculitis is a disease with a broad spectrum of clinical symptoms from life threatening fulminant symptoms to relatively minor skin erythema [1,2]. There has been significant progress in understanding of pathogenesis, however, the precise nature of the triggering events remains still elusive. The etiology is multifactorial including genetic factors and environmental etiologies (ultraviolet light, infections, toxins, drugs, and metal allergies) [1-5].

In general, the development of secondary systemic vasculitis after orthopaedic operations is uncommon [1,6,7]. However, specific surgical procedures have been more commonly associated with the disease comparing to others, such as total hip arthroplasties with metal on metal bearing surfaces; and this has been attributed mainly to allergic reactions against nickel or cobalt ions which are released from wear particles [6-8]. Systemic vasculitis after spinal instrumentation is very rare [9,10]. Identification of possible etiologies that cause the specific immunologic reaction is sometimes difficult and controversial [1-5].

The purpose of this paper was to highlight the difficulties in diagnosis and management of a case with a history of scoliosis -which was corrected with posterior instrumented fusion- and developed systemic vasculitis. The diagnostic dilemma was related to the cause of such an immunologic over-reaction. The main questions were if this case was associated with a late infection or allergic reaction to titanium-alloy implants, and to present a diagnostic and treatment work-up for these cases.

## Case Report

**Figure 1:**
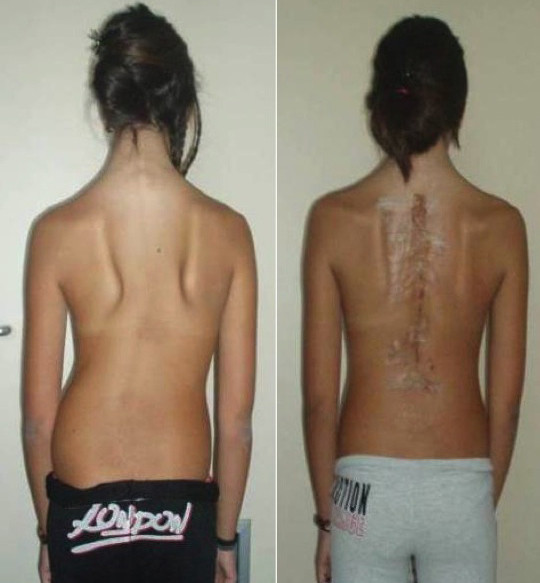
**Preoperative (left) and postoperative (right) clinical images showing the correction of the scoliotic deformity**. However, at the fourth postoperative day there was a wound dehiscence that was undergone debridement and re-suturing.

A 14 year-old girl with a history of idiopathic scoliosis underwent surgical correction of scoliotic deformity with posterior spinal instrumentation and fusion using a spinal implant containing titanium alloy (T1-641-4VASTMF-130). The postoperative period was uncomplicated in general; except for a wound dehiscence at the fourth day post surgery that was repaired surgically with debridement and immediate skin closure (Figure 1).

**Figure 2:**
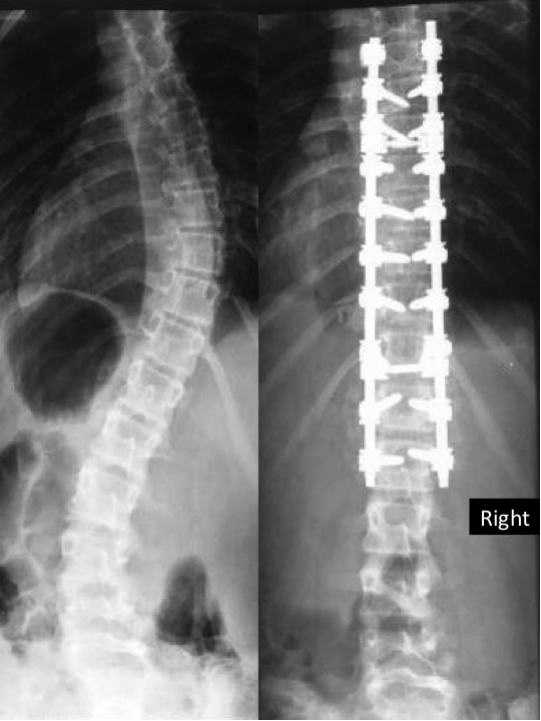
**Standing postero-anterior preoperative (left) and postoperative (right) radiographic images showing correction of scoliotic deformity**.

During the scheduled follow-up visits at 1st and 3rd month the clinical and radiographic image of the patient was normal. Surgical wound was completely healed with no signs of inflammation. Correction of scoliotic deformity both in frontal and sagittal planes has been achieved and patient was satisfied with overall outcome (Figures 1 &2).

**Figure 3:**
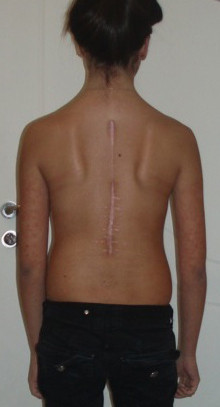
**Clinical image of the patient, five months postoperatively, showing the development of a diffuse erythema that was evident mostly in the upper limbs and the trunk**.

Five months postoperatively, the parents seeked pediatric consultation because their child developed macroscopic hemuresis (blood in urine) and diffuse erythema (Figure 3). The patient was in good general condition. Laboratory studies on admission revealed that the white blood cell count was 9100/mL; granulocytes, 39%; monocytes, 6%; lymphocytes, 19%; and eosinophils, 36%. The hematocrit was 35.5% and the platelet count was 185 × 10^3^/mL. C-reactive protein (CRP) was 5.0 mg/dL (0.7 to 1.7 mg/dL). Erythrocyte Sedimentation Rate was 75 mm.

Modest elevations in transaminases and bilirubin were noted: total bilirubin was 1.4/dL, AST 59 and ALT 34. Renal biochemistry showed no abnormalities: blood urea nitrogen was 11 mg/dL and creatinine 0.8 mg/dL. Urine trace was positive for bilirubin, ketones, and protein. The urinary sediment contained 8 RBCs per high-power field and 7 WBCs per high-power field. No cellular casts were identified.

Complement levels were normal. Antinuclear antibodies, rheumatoid factor, anti-neutrophil cytoplasmic antibodies, antibodies to glomerular basement membrane were normal. Hepatitis B surface antigen, hepatitis C antibody, and HIV antibodies were negative.

Differential diagnosis included late periprosthetic infection, low virulence viral or bacterial infection unrelated to surgery, allergic reaction to metal implants or to other envinmental agents, and toxicity. Skin patch testing for metal hypersensitivity was strongly positive for titanium and nickel, supporting the role of the titanium implants in the development of secondary systemic vasculitis. The patient received corticosteroids systematically (hydrocortisone 10 mg) for 6 months, leading to total recess of erythema, hemuresis and proteinuria. Orthopedic surgery was consulted to consider removal of the spinal implants. After weighing the risks and benefits of the procedure, the titanium prosthesis was not removed, because spinal fusion was premature and early removal of instrumentation could inevitably lead to loss of reduction.

**Figure 4:**
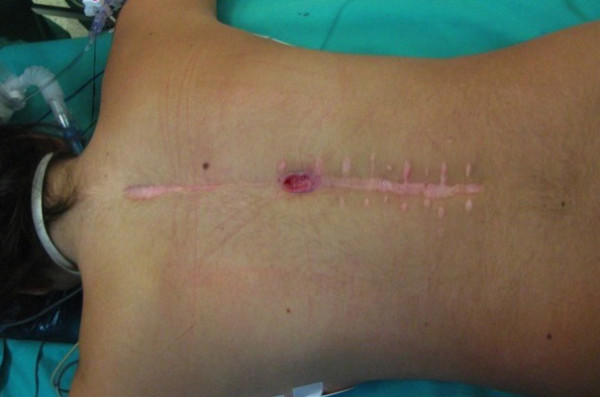
**A palpable mass close to surgical wound and a small skin dehiscence of the surgical scrar was developed 1 month post corticosteroid cessation**.

**Figure 5:**
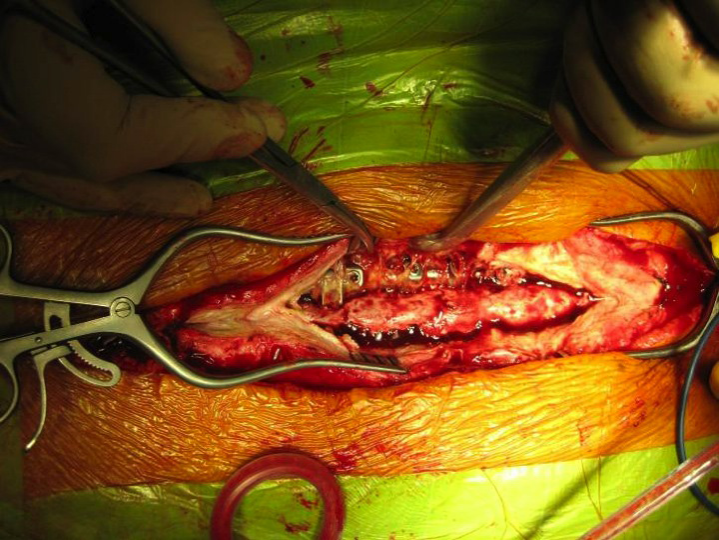
**Intraoperative image showing the present of pus surrounding the spinal implants**.

**Figure 6:**
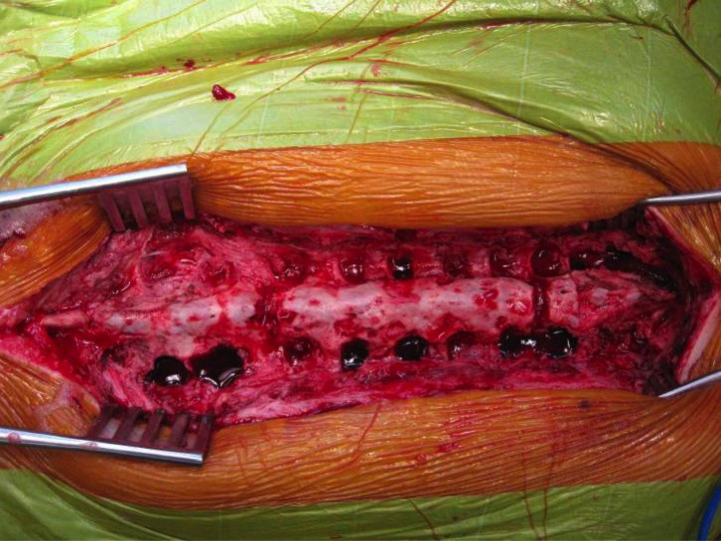
**Meticulous surgical debridement and one stage revision of posterior spinal instrumentation in order to retain correction of scoliotic deformity**.

However, 1 month post corticosteroid cessation a palpable mass close to surgical wound and a small skin dehiscence of the surgical scar was developed (Figure 4). A soft tissue ultra-sonography showed the presence of cystic formation 3 × 6 cm within the muscle layers of the thoraco-lumbar region and close to the spinal implants. Surgical debridement was decided which revealed the presence of pus with gram positive staining (Figure 5). There was some callus formation over the decorticated and grafted posterior elements as well as the osteotomized facet joints which was proved soft when we tried to remove the cross-links. Notwithstanding there was some minimal "elastic" motion of the spine after removing the metalwork. Therefore, one stage revision of posterior spinal instrumentation was performed as pus collection was deep and very close to the spinal implants (Figure 6). Although we were prepared for revision of the instrumentation with titanium and nickel-free implants, the presence of pus in the surgical field made evident that the etiology of vasculitis was a late infection and not a metal allergy. Therefore, we proceeded to a meticulous surgical debridement and re-implantation of titanium spinal implants which are associated with decreased rates of infection comparing to stainless steel. Intraoperative sample cultures were positive to Staphylococcus aureus. Intravenous antibiotics (were administered for three weeks followed another 3 week period of oral administration.

### Postoperative Course

The postoperative recovery was uneventful and the patient had complete resolution of symptoms. Neurologic examination was normal at the 6-week postoperative visit and the x-ray imaging in postero-anterior and lateral views showed no loss of initial reduction or implant loosening. Twenty-four post revision surgery, the patient is now free of symptoms without any signs of recurrence of either allergy or infection. There is no need for pain medication and the patient is back to daily activities without restrictions.

## Discussion

We presented the case of a 14-year-old female with a secondary systemic vasculitis few months after posterior spinal fusion due to idiopathic scoliosis aiming to highlight the difficulties during the diagnostic process. Differentiating possible etiologies for secondary systemic vasculitis is sometimes confounding. Symptoms and laboratory findings may be misleading and decision-making could be controversial and problematic [1-5]. Herein, we faced a significant problem in distinguishing the possible causative factor that triggered the immunologic over-reaction and caused the systematic vasculitis. Due to lack of precedence, we had entertained 2 possibilities for the present clinical dilemma: a low-grade chronic infection; and delayed immune reaction to the metal of implant. Although initial clinical and laboratory findings were in favor of an allergenic etiology, we finally identified the presence of periprosthetic spinal infection. However, the question if the infection triggered the systemic immune reaction or the immune hyper-reactivity and possible formation of seroma resulted in contamination and periprosthetic infection remained unanswered.

Systemic vasculitis is a disease with broad spectrum of clinical symptoms. The severity ranges widely from life threatening fulminant conditions to relatively minor skin disease [1-5]. The etiology is clearly multifactorial; among the potential influences on disease expression are genetic factors (HLA and others), ultraviolet light, infections, toxins, drugs, and allergies [1-5]. In our case, we could identify two potential contributing factors for the development of a secondary systematic reaction: late infection which is not unusual and metal allergy to titanium or titanium alloy components, that is relatively rare [11]. However, it is likely that cases involving implant-related metal sensitivity have been underreported because of the difficulty of diagnosis [1,11]

Metal hypersensitivity has been associated mainly with hip joint replacement recipients with metal on metal bearing surfaces. This could be attributed to exposure to degradation products (i.e wear particles of metal on metal bearing surfaces) that mediate t immunologic effects and/or cell toxicity [6-8]. Spinal implants are static load-bearing devices subjected to micromotion at least until fusion is achieved. The long instrumentations for spinal deformity involve many couplings of screws, rods and interconnecting devices, all with a potential to fretting corrosion. Therefore, spinal instrumentation can cause metal ion release from fretting corrosion with elevated levels in body fluids [10]. This has been demonstrated by several studies on metal ion levels in spinal instrumentation [12,13]. Moreover, titanium particulate debris at the level of a spinal arthrodesis could elicit a cytokine-mediated particulate-induced response favoring pro-inflammatory infiltrates, increased expression of intracellular tumor necrosis factor-alpha, increased osteoclastic activity, and cellular apoptosis, as shown in an animal model by Cunningham et al. [14] In the clinical setting, the presence of titanium particulate debris, secondary to motion between spinal implants could serve as the impetus for late-onset inflammatory-infectious complications and long-term osteolysis of an established posterolateral fusion mass [14].

Patients presenting with signs of a systemic reaction should be evaluated for sensitivity. Assessment of hypersensitivity has historically been conducted in vivo by skin testing (i.e. patch testing or intradermal testing) [15-17] and in vitro by leukocyte migration inhibition testing (termed LIF or MIF testing) [18,19] and MELISA (memory lymphocyte immunostimulation assay) [20-22]. MELISA reactivity is directly dependent on lymphocyte concentration; the higher the lymphocyte concentration per test, the stronger the reactivity [20-22]. While in vivo testing protocols and commercial kits do exist, there is continuing concern about the applicability of skin testing to the study of immune responses to implants, particularly since there is a lack of knowledge about and availability of appropriate challenge agents [15-17]. Basketter et al. [23] and Okamura et al. [24], suggested specific titanium salts for testing in case of suspected titanium allergy. Although the utility of migration inhibition assays in various clinical settings has been demonstrated [18,19], only Merritt et al. [25] have applied leukocyte migration testing to assess biocompatibility of implanted devices. In our case, there was no availability of in vitro leukocyte migration inhibition testing.

Except for the clinical scenario of immune hypersensitivity due to metal allergy, other potential diagnoses or etiologies should always be considered. Delayed immune response due to sub-acute, low virulence infection is one possible cause [1,26]. A complete diagnostic work up should be performed, including cultures of urine and blood samples, chest x-rays, blood count, and evaluation of erythrocyte sedimentation rate and C-reactive protein levels [27]. Surgical wound should always be assessed both clinically for signs of inflammation, fistulas or fluid collections and with imaging studies (MRI, CT, ultrasonography) which usually indicate the presence periprosthetic infection [27]. However, even in cases with loud clinical symptomatology and increased suspicion for the presence of infection, there should be a great level of awareness. Aseptic fistulas might become the gate of infection appeared in the body of the patients, whose implants worked improperly. The patients who suffered the complication were allergic to the metals included in the implant [1,26]. This could be the case in our patient. Increased immune reaction due to metal allergy could induce a secondary formation of seroma, dehiscence of surgical wound and eventually contamination and establishment of a periprosthetic infection; and vice versa, a primary periprosthetic infection could cause an over-reaction of the immune system in the form of systemic vasculitis.

Other possible etiologic factors of systemic immunologic response should probably be excluded using the appropriate laboratory exams. Antinuclear antibodies, rheumatoid factor, anti-neutrophil cytoplasmic antibodies, antibodies to glomerular basement membrane and complement levels should be assessed in order to exclude immunologic pathologies [4,5] Hepatitis B surface antigen, hepatitis C antibody, and HIV antibodies should be evaluated in order to excluded immune-hyper-reactivity due to viral infection [28]. In our case all above mentioned laboratory exams were within normal values.

Removal of a device that has served its function should be considered, since removal may alleviate the symptoms. However, in the case of instrumented posterior spinal fusion there should always be taken into account the great possibility of loss of reduction if the implants are removed too early [29]. The severity of systemic symptoms should be weighed over the potential loss of reduction. In a retrospective analysis of 45 cases with late developing infection after instrumented posterior spinal fusion for scoliosis, Muschic et al [30] suggested re-instrumentation after implant removal in order to reduce the loss of correction.

In conclusion, the development of a secondary systemic vasculitis after orthopaedic surgeries and especially spinal instrumented procedures is a generally rare. Metal hypersensitivity and periprosthetic infection are well-recognized etiologic factors. Significant difficulties in evaluating suspected titanium hypersensitivity still exists, as there are no standardized valid patch tests and leukocyte migration testing is not broadly available. The case reports and studies published so far reflect the diagnostic uncertainties in evaluating suspected titanium hyper-reactivity and show that this condition is uncommon. Investigation for the presence of late infections should always be performed as they could be directly or indirectly related to triggering of immune hypersensitivity and systemic reaction.

## Consent

Written informed consent was obtained from the mother of the patient for publication of this case report and any accompanying images. A copy of the written consent is available for review by the Editor-in-Chief of this journal.

## List of abbreviations

CRP: C-Reactive Protein; RBC: Red Blood Cells; WBC: White Blood Cells; HIV: Human Immunodeficiency Virus; MRI: Magnetic Resonance Imaging; CT: Computed Tomography; LIF: leukocyte migration inhibition testing; MELISA: memory lymphocyte immunostimulation assay.

## Competing interests

We declare that there are no financial and non-financial competing interests related to the publication of this manuscript. Specifically, in the past five years, we have not received any reimbursements, fees, funding, or salary from an organization that may in any way gain or lose financially from the publication of this manuscript, either now or in the future. We do not hold any stocks or shares in an organization that may in any way gain or lose financially from the publication of this manuscript, either now or in the future. We do not hold or apply for any patents relating to the content of the manuscript. There are no non-financial competing interests (political, personal, religious, ideological, academic, intellectual, commercial or any other) to declare in relation to this manuscript.

## Authors' contributions

VIS and KS drafted the manuscript. EA, TB and PP reviewed the literature. KS, KS and CB critically reviewed the manuscript. All authors read and approved the final manuscript.
